# The PHD2 oxygen sensor paves the way to metastasis

**DOI:** 10.18632/oncotarget.6216

**Published:** 2015-10-21

**Authors:** Anna Kuchnio, Mieke Dewerchin, Peter Carmeliet

**Affiliations:** Laboratory of Angiogenesis and Neurovascular Link, Vesalius Research Center, VIB - KU Leuven, Campus Gasthuisberg O&N4, Herestraat 49-912, B-3000, Leuven, Belgium

**Keywords:** breast cancer, metastasis, cancer associated fibroblast, oxygen sensor, vessel normalization

Breast cancer is the most frequent cancer and remains the second leading cause of cancer death in women. Metastatic relapse is a main cause of this high mortality. Understanding the mechanisms that control metastasis is therefore pivotal for the design of improved and safe breast cancer treatment regimen.

Hypoxia is a characteristic feature of most solid tumors, including breast cancer, and is a strong stimulus of tumor cell invasion and metastasis. Hypoxia signaling regulates nearly every step of the metastatic cascade, including epithelial-to-mesenchymal transition, intravasation, survival in the circulation, formation of the pre-metastatic niche, and growth from micro- to macro-metastatic lesions. Furthermore, hypoxic tumors display lower sensitivity to treatment, leading to poor prognosis. Prolyl-hydroxylases (PHD1-3) are oxygen sensors involved in hypoxia regulation. Despite the crucial role of egl-9 family hypoxia-inducible factor 1 (EGLN1, best known as PHD2) as an oxygen sensor, its role in tumor growth and metastasis in general and of breast cancer in particular, remains debated. Previous studies from us and other research teams on PHD2 in cancer highlighted different possible roles of PHD2 that may be cell-type dependent. On the one hand, we previously demonstrated that haplodeficiency of *Egln1* selectively in *endothelial cells (ECs*) reduced metastasis without affecting tumor growth, by normalizing the abnormal tumor vessels and reducing tumor cell intravasation [1, 2]. Using transplantable tumor models, others reported that silencing of PHD2 in *cancer cells* either increased or decreased tumor growth with different underlying mechanisms [3-5]. Dissection of the role of PHD2 in conditions that allow the evaluation of cell-intrinsic effects as well as the impact of bidirectional tumor / stroma cross-talk, remains strongly warranted. This is particularly relevant in light of pharmacological PHD2 blockade, which would target PHD2 in all cells inside the tumor. Furthermore, the studies mentioned above only used transplantable tumor models. The role of PHD2 in breast cancer using a clinically more relevant model of spontaneously arising breast cancer thus remained undefined.

**Figure 1:**
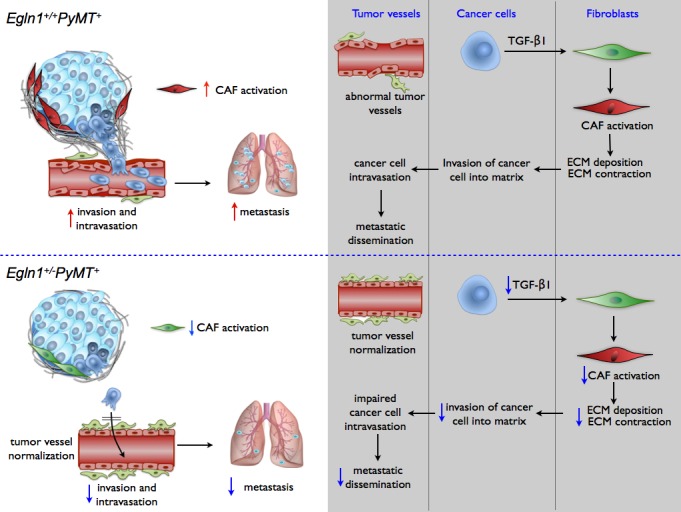
Dual role of PHD2 (Egln1) in promoting breast cancer metastasis, involving CAF-mediated deposition of collagen tracks and abnormalization of tumor vessels

We therefore recently investigated the role of PHD2 using the spontaneously arising PyMT-oncogene driven breast cancer model (MMTV-PyMT model) and intercrossed this transgenic line with mice with heterozygous gene deficiency of *Egln1* (*Egln1*^+/−^ mice; further named *Egln1*^+/−^*PyMT*^+^ mice upon intercross with the the PyMT line) [6]. We observed that tumor growth was unaffected, but metastasis and intravasation were markedly reduced in *Egln1*^+/−^*PyMT*^+^ mice as compared to control mice (PyMT mice intercrossed with *Egln1* wild type mice; further named *Egln1*^+/+^*PyMT*^+^ mice). Applying genetic strategies *in vivo* and *in vitro*, we showed that this reduction in metastasis and intravasation could be ascribed to two independent mechanisms (Figure 1). First, we found that global “genetic targeting” of *Egln1* in the entire tumor in *Egln1*^+/−^*PyMT*^+^ mice induces tumor vessel normalization characterized by a tighter endothelial lining, improved pericyte coverage, and improved perfusion, similar to selective *Egln1* haplodeficiency in ECs in xenograft models [1, 2]. Secondly, reduction in metastasis was also attributable to reduced activation of cancer-associated fibroblasts (CAFs). As compared to *Egln1*^+/+^*PyMT*^+^ tumors, *Egln1*^+/−^*PyMT*^+^ tumors contained fewer activated CAFs, which deposited less cross-linked collagen matrix and contracted the collagen matrix less. These processes are known to induce cancer cell invasion. We showed that reduced CAF activation was independent of the PHD2 level in fibroblasts, but reliant on the level of PHD2 in cancer cells. *Egln1* haplodeficiency in cancer cells lowered the release of TGF-β1 and diminished the differentiation of normal fibroblasts to activated CAFs (Figure 1) [6].

Our findings invite additional lines of investigation, both with respect to the underlying regulatory mechanisms and, importantly, to clinical translation. For instance, from a conceptual perspective, it is puzzling that inactivation of PHD2 by hypoxia in the tumor microenvironment suppresses the pro-metastatic activity of CAFs, given the vast literature that hypoxia promotes metastasis. The precise pathophysiological purpose of this phenomenon remains to be elucidated. Regardless however, from a therapeutic perspective, blocking this CAF-dependent pro-metastatic activity of PHD2 might offer novel opportunities to suppress cancer cell dissemination. Indeed, we provided genetic evidence that global *Egln1* haplodeficiency from the start of tumorigenesis is not only well tolerated, but also reduces metastatic disease. Interestingly, *Egln1* blockade initiated at the later stages, when invasive adenoma is already present, is also sufficient to reduce metastasis [6]. This implies that administration of a pharmacological PHD2 blocker, which would inhibit PHD2 in both cancer and stromal cells, might be therapeutically considered to prevent / minimize metastatic disease. Needless to say that the applicability and generality of such approaches requires extensive further exploration in breast cancer models and other models of spontaneous cancer.

The notion that cancer cells exploit interactions with stroma cells to create a local environment beneficial for their own growth or dissemination is becoming increasingly clear. This was previously also shown in hematological malignancy, bone metastases and medullablastoma [7]. The PHD2-mediated breast cancer cell - CAF interaction revealed in our study represents an additional example of such reciprocal, tumor-promoting interaction.
